# Noise Reduction Method of Underwater Acoustic Signals Based on Uniform Phase Empirical Mode Decomposition, Amplitude-Aware Permutation Entropy, and Pearson Correlation Coefficient

**DOI:** 10.3390/e20120918

**Published:** 2018-11-30

**Authors:** Guohui Li, Zhichao Yang, Hong Yang

**Affiliations:** School of Electronic Engineering, Xi’an University of Posts and Telecommunications, Xi’an 710121, China

**Keywords:** underwater acoustic signals, uniform phase empirical mode decomposition, amplitude-aware permutation entropy, Pearson correlation coefficient, noise reduction

## Abstract

Noise reduction of underwater acoustic signals is of great significance in the fields of military and ocean exploration. Based on the adaptive decomposition characteristic of uniform phase empirical mode decomposition (UPEMD), a noise reduction method for underwater acoustic signals is proposed, which combines amplitude-aware permutation entropy (AAPE) and Pearson correlation coefficient (PCC). UPEMD is a recently proposed improved empirical mode decomposition (EMD) algorithm that alleviates the mode splitting and residual noise effects of EMD. AAPE is a tool to quantify the information content of nonlinear time series. Unlike permutation entropy (PE), AAPE can reflect the amplitude information on time series. Firstly, the original signal is decomposed into a series of intrinsic mode functions (IMFs) by UPEMD. The AAPE of each IMF is calculated. The modes are separated into high-frequency IMFs and low-frequency IMFs, and all low-frequency IMFs are determined as useful IMFs (UIMFs). Then, the PCC between the high-frequency IMF with the smallest AAPE and the original signal is calculated. If PCC is greater than the threshold, the IMF is also determined as a UIMF. Finally, all UIMFs are reconstructed and the denoised signal is obtained. Chaotic signals with different signal-to-noise ratios (SNRs) are used for denoising experiments. Compared with EMD and extreme-point symmetric mode decomposition (ESMD), the proposed method has higher SNR and smaller root mean square error (RMSE). The proposed method is applied to noise reduction of real underwater acoustic signals. The results show that the method can further eliminate noise and the chaotic attractors are smoother and clearer.

## 1. Introduction

The processing and analysis of underwater acoustic signals play a significant role in the area of military and marine exploration. Due to the influence of the accuracy of measuring instrument and the interference of marine environment, underwater acoustic signals are often contaminated by various noises. The true physical characteristics of underwater acoustic signals are masked, consequently, it is necessary to denoise [1,2]. Underwater acoustic signals are nonlinear, non-Gaussian, and non-stationary chaotic signals [3]. In the time-frequency domain, it has strong broadband properties and pseudo-randomness. This causes the frequency band of the signal to partially or completely overlap with the frequency band of the noise. Therefore, the conventional filtering method is not suitable for noise reduction of underwater acoustic signals [4,5]. In order to effectively suppress additive noise, wavelet analysis, local projection, and singular spectral analysis have been applied to underwater acoustic signals processing, but these methods have certain limitations to different degrees [6,7]. As an example, the wavelet transform has the limits of the selection of the appropriate wavelet basis, decomposition level, and wavelet threshold; the local projection method has the limits of the selection of neighborhood radius; the singular spectral analysis method needs to solve the problem of principal component selection.

Empirical mode decomposition (EMD) [8] provides a new idea of underwater acoustic signals processing. It can decompose the original signal into a series of intrinsic mode functions (IMFs). Each oscillation mode of the original signal represented an IMF. However, EMD has some problems, such as mode mixing, residual noise, and endpoint effects. To overcome these shortcomings, a multitude of methods have been put forward including the masking signal EMD (MS-EMD) [9], ensemble empirical mode decomposition (EEMD) [10], extreme-point symmetric mode decomposition (ESMD) [11], complete ensemble empirical mode decomposition with adaptive noise (CEEMDAN) [12], nonlinear mode decomposition (NMD) [13], and improved empirical mode decomposition (IEMD) [14]. Both MS-EMD and EEMD are based on a similar idea: adding certain disturbances to the original signal in order to obtain a perturbed signal with a more uniform distribution of extrema. For example, MS-EMD adds a sinusoidal signal, and EEMD adds Gaussian white noise. Based on EMD, ESMD optimizes the external envelope interpolation method to internal pole-symmetric interpolation, thus, it improves the mode mixing problem. Wang et al. proposed the uniform phase empirical mode decomposition (UPEMD) algorithm [15], which selected sinusoidal functions with uniform phase distribution as perturbed signals, and significantly alleviated the mode splitting and residual noise effects of MS-EMD and EEMD simultaneously.

Shannon entropy is used to quantify the information content of time series. In recent years, researchers have also come up with the approximate entropy (ApEn) [16], fuzzy entropy (FE) [17], sample entropy (SE) [18], and permutation entropy (PE) [19]. PE is based on permutation patterns or order relations among values of a signal, and it has the advantages of simple calculation and relative robustness to observational and dynamical noise. Although the calculation efficiency is high, PE does not consider the amplitude information of the signal. To overcome this problem, many researchers have proposed modified algorithms, such as weighted-permutation entropy (WPE) [20], permutation min-entropy (PME) [21], and amplitude-aware permutation entropy (AAPE) [22]. AAPE is put forward in 2016, which takes into account the amplitude information of the signal and has a wider application prospect.

Recently, an ocean of noise reduction methods based on signal decomposition algorithms and Shannon entropy have been developed and used in different fields, such as acoustic signal [23,24], hydropower unit vibration signal [25], bearing vibration signal [26], medical signal [27], wind speed prediction [28], and so on. Xiao et al. [29] proposed a fault denoising and feature extraction method of rolling bearing based on NMD and continuous wavelet transform (CWT). Zhan et al. [30] used CEEMDAN and FE to denoise the shock signal. The relevant modes (noisy signal modes and useful signal modes) can be identified by the difference between the FE of the new waveform and the next adjacent new waveform. Li et al. [31] proposed a noise reduction method of underwater acoustic signals based on CEEMDAN, mutual information, PE and CWT. Ma et al. [32] proposed EEG signal denoising method based on EEMD and cosine similarity (CS). The similarity between IMF and the original signal is calculated by CS. Then, the IMFs are divided into noise-dominated and signal-dominated part by finding the latter location of the first minimal value of CS curve. Bi et al. [33] used EEMD, robust independent component analysis (RobustICA) and CWT to study the blind source separation and noise source identification of gasoline engines. As far as we know, UPEMD and AAPE have not been used to solve the problem of underwater acoustic signals.

In this paper, a noise reduction method of underwater acoustic signals based on UPEMD, AAPE and Pearson correlation coefficient (PCC) is proposed. We used UPEMD to decompose the original signal into a series of IMFs. According to the threshold of AAPE, high-frequency IMFs and low-frequency IMFs can be distinguished effectively. It is considered that all low-frequency IMFs are useful IMFs (UIMFs), and then PCC is used to determine whether the high-frequency IMF with the smallest AAPE is a UIMF. Finally, the noise reduction can be realized by reconstructing all UIMFs. The structure of this paper is as follows: Section 2 is the basic theory of UPEMD, AAPE, and PCC; in Section 3, the proposed noise reduction method of underwater acoustic signals and evaluation criteria is introduced; in Section 4 and Section 5, the proposed method is applied to chaotic signal and underwater acoustic signals respectively. The conclusion is given in Section 6.

## 2. Basic Theory

### 2.1. Uniform Phase Empirical Mode Decomposition (UPEMD)

The main purpose of the EMD algorithm is to decompose the original signal f(t) into a series of IMFs that characterize the time scale, which can be expressed below:

(1) $$f(t)=\sum IMF_{i}(t)$$

The specific steps of EMD are briefly summarized as follows:

Step 1. Connect the local maxima/minima of x(t) to obtain the upper/lower envelope using the cubic spline.
Step 2. Derive the local mean of envelope, m(t), by averaging the upper and lower envelopes.
Step 3. Extract the temporary local oscillation h(t)=x(t)−m(t).
Step 4. If h(t) satisfies some predefined stoppage criteria [8], h(t) is assigned as an IMF noted as $c_{m}(t)$ where m is the IMF index. Otherwise set x(t)=h(t) and repeat Step 1 to Step 3.
Step 5. Compute the residue $r_{m}(t)=x(t)-c_{m}(t)$.
Step 6. Set $x(t)=r_{m}(t)$ and repeat Step 1 to Step 5 to extract the next IMF.

Note that the fixed sifting number $n_{s}$ is one of the stoppage criteria of EMD [8].

#### 2.1.1. The Masking Signal EMD (MS-EMD)

UPEMD is based on the idea of MS-EMD. Before introducing the concept of UPEMD, we first introduce MS-EMD. To alleviate the residual noise effects of EMD, Deering et al. proposed MS-EMD in 2005 [9]. The masking EMD uses a sinusoid signal w(t) as the assisted disturbance with its frequency $f_{w}$ no less than that of the highest frequency component of data. Then EMD is applied to decompose the signal x(t) perturbed by w(t) into two IMFs. The same process is repeated by applying EMD to the signal perturbed by −w(t). Finally, the average of the two resultant sets of IMFs is identified as the final result [9]. Let $E_{m}(\cdot )$ be an operator, which produces the mth IMF decomposed by EMD.

The specific steps of MS-EMD are summarized as follows:

Step 1. A masking signal is constructed according to the frequency information of the original signal x(t):
  (2) $$w(t;\theta )=\varepsilon \cos(2\pi f_{w}t+\theta )$$
Step 2. Compute $c_{1}^{+}=E_{1}\left(x(t)+w(t;\theta )\right)$; Similarly, compute $c_{1}^{-}=E_{1}\left(x(t)+w(t;\theta +\pi )\right)$.
Step 3. Obtain IMF1 by $c_{1}=\frac{c_{1}^{+}+c_{1}^{-}}{2}$, and IMF2 by $c_{2}=x-c_{1}$.

The disturbance amplitude ε and phase θ of the assisted sinusoid signal can be preset according to the relevant rules of the MS-EMD. The specific method is described in [9].

#### 2.1.2. The Two-Level EMD (2L-UPEMD)

MS-EMD uses two sinusoidal signals to eliminate residual noise, but the effect is underdeveloped. Wang et al. [15] pointed out that residual noise can be minimized by searching for all possible phases. Based on this idea, 2L-UPEMD is proposed. Denote the number of phases as $n_{p}$ with $n_{p}\in N$. Let these $n_{p}$ phases be uniformly distributed in the interval [0,2π]. Then the phase $\theta_{k}$ in the kth realization in Equation (2) is calculated as:

(3) $$\theta_{k}=2\pi (k-1)/n_{p},k=1,2,\ldots ,n_{p}$$

The specific steps of 2L-UPEMD are summarized as follows:

Step 1. Assign $T_{w}(=1/f_{w})$, ε and $n_{p}$.
Step 2. Based on Equations (2) and (3), calculate the perturbed signal:
  (4) $$w(t;\theta_{k})=x(t)+\varepsilon \cos\left(2\pi \left(f_{w}t+\frac{k-1}{n_{p}}\right)\right)$$
Step 3. Perform EMD to obtain two IMFs, $c_{k,m}(t)=E_{m}\left(x(t)+w(t;\theta_{k})\right),m=1,2$.
Step 4. Repeat Step 2 to Step 3 for k=1 to $n_{p}$.
Step 5. Obtain the resultant IMF1 and IMF2 as $c_{m}(t)=\frac{1}{n_{p}}\sum_{k}c_{k,m}(t)$.

The observation formula shows that when $n_{p}=2$, 2L-UPEMD is MS-EMD.

#### 2.1.3. The Multi-Level UPEMD

As mentioned in Section 2.1.2, 2L-UPEMD can decompose the signal into two IMFs. The IMF1 is identified as the resultant IMF1, and IMF2 is identified as the new signal. The same method is applied recursively to extract the resultant IMFs at lower frequencies [15].

Assuming a time series of n samples in length. The masking frequency $f_{w}$ is predetermined by taking the dyadic property of EMD that acts as an adaptive dyadic filter bank for the decomposition of the white noise with $n_{s}=10$ [10]. The number of resultant IMFs is approximately equal to $n_{imf}=\log_{2}n$. The period with index m is determined as $T_{w}=2^{m}$, $m=1,2,\ldots ,\log_{2}n$. Let $U_{m}(\cdot )$ be an operator, which produces the mth IMF decomposed by 2L-UPEMD.

The specific steps of multi-level UPEMD are summarized as follows:

Step 1. Assign $n_{p}$, set $n_{imf}=\log_{2}n$ and initial residue: $r_{0}(t)=x(t)$.
Step 2. Set $\varepsilon_{m}=\varepsilon_{0}\cdot std(r_{m-1}(t))$, where std stands for the standard deviation; and ${\left(T_{w}\right)}_{m}=2^{m}$.
Step 3. Perform the 2L-UPEMD to obtain the IMF $c_{m}(t)$, that is $c_{m}(t)=U_{1}\left(r_{m-1};n_{p},\varepsilon_{m},{\left(T_{w}\right)}_{m},n_{s}\right)$.
Step 4. Calculate residue $r_{m}(t)\leftarrow r_{m-1}(t)-c_{m}(t)$.
Step 5. Repeat Step 2 to Step 5 for m=1 to $n_{imf}$ to extract all IMFs.

Note that the UPEMD used in this paper is the multi-level UPEMD. The disturbance amplitude $\varepsilon_{0}$ is usually empirically chosen over the range of $\varepsilon_{0}\approx 0.1\sim 1.0$, $n_{p}$ is the maximum number of phases allowed in each IMF. According to the default values recommended in [15], $\varepsilon_{0}$ and $n_{p}$ are set to 0.2 and 8, respectively. The Matlab codes of UPEMD are available at http://in.ncu.edu.tw/mzlo/drLo.html.

### 2.2. Amplitude-Aware Permutation Entropy (AAPE)

PE is a computational time series complexity method proposed by Bandt and Pompe. It has the characteristics of simple, fast and robust calculation methods [19]. Assuming a time series of N samples in length, that is y(n)={y(1),y(2),…,y(N)}, the first step to compute this entropy is to form N−d+1 vectors of size d samples (where d denote the embedding dimension), such that $\begin{array}{l} Y_{d}(i)=\left\{y(i),x(i+1),\ldots ,y(i+d-1)\right\} \end{array}$, for $\begin{array}{l} 1\leq i\leq N-d+1 \end{array}$. Next, an ordinal pattern is associated with each vector $\begin{array}{l} Y_{d}(i) \end{array}$, which is defined as the permutation $\begin{array}{l} \varphi_{i}=\left\{r_{0},r_{1},\ldots ,r_{d-1}\right\} \end{array}$ of $\begin{array}{l} \left\{0,1,\ldots ,d-1\right\} \end{array}$ that fulfills $y(i+r_{0})\leq y(i+r_{1})\leq \ldots \leq y(i+r_{d-2})\leq \begin{array}{l} y(i+r_{d-1}) \end{array}$. Thus, d! different ordinal patterns, referred to as $\pi_{k}$, can be obtained from vectors $Y_{d}$. For instance, six different ordinal patterns can be considered for d=3, such as $\pi_{1}=\left\{0,1,2\right\}$, $\pi_{2}=\left\{0,2,1\right\}$, $\pi_{3}=\left\{1,0,2\right\}$, $\pi_{4}=\left\{1,2,0\right\}$, $\pi_{5}=\left\{2,0,1\right\}$, and $\pi_{6}=\left\{2,1,0\right\}$ [34]. For each $\pi_{k}$, $p(\pi_{k})$ represents the relative frequency as follows:

(5) $$p(\pi_{k})=\frac{\sum_{i=1}^{N-d+1}\delta (\pi_{k},\varphi_{i})}{N-d+1}$$

where δ(u,v) is the Kronecker delta function modified to work with patterns, such that:

(6) $$\delta (u,v)=\left\{ \begin{array}{l} 1,\;\mathrm{if}\;u(i)=v(i),\mathrm{for}\;\mathrm{every}\;i=1,2,\ldots ,d;\\ 0,\;\mathrm{for}\;\mathrm{otherwise}. \end{array}\right.$$

Finally, the normalized PE is computed as follows:

(7) $$PE(d)=-\frac{1}{\ln(d!)}\sum_{k=1}^{d!}p(\pi_{k})\cdot \ln(p(\pi_{k}))$$

When $p(\pi_{k})=\frac{1}{d!}$, the PE reaches the maximum value 1. PE denotes a random degree of the time series y(n). The smaller the PE, the more regular the time series; otherwise, the more random the time series.

PE only considers ordinal structure of patterns, the amplitude of each sample is discarded [34]. For instance, vectors $Y_{d}(i)=\left\{5,10,2\right\}$ and $Y_{d}(j)=\left\{11,26,8\right\}$ produce the same ordinal pattern $\pi_{k}=\left\{2,0,1\right\}$, thus, their PE values are equal. Azami et al. proposed the amplitude-aware permutation entropy (AAPE) for the above problems. Briefly, the repetition probability of each pattern $\pi_{k}$ is estimated by considering its relative frequency, as well as the average absolute (AA) and relative amplitudes (RA) associated with the vectors $Y_{d}$. For a specific vector $Y_{d}(i)$, the amplitudes can be expressed as follows:

(8) $$AA_{i}=\frac{1}{d}\sum_{l=1}^{d}\left|y(i+l-1)\right|$$

(9) $$RA_{i}=\frac{1}{d-1}\sum_{l=2}^{d}\left|y(i+l-1)-y(i+l-2)\right|$$

Then, the occurrence probability of $\pi_{k}$ can be expressed as:

(10) $$p^{*}(\pi_{k})=\frac{\sum_{i=1}^{N-d+1}\delta (\pi_{k},\varphi_{i})\cdot \left(\alpha \cdot AA_{i}+(1-\alpha )\cdot RA_{i}\right)}{\sum_{i=1}^{N-d+1}\alpha \cdot AA_{i}+(1-\alpha )\cdot RA_{i}}$$

where α is an adjusting coefficient, for α∈[0,1], it makes the AAPE algorithm more flexible. In this paper, we set α=0.5 according to the suggestion in [22]. Finally, the normalized AAPE is computed as follows:

(11) $$AAPE(d)=-\frac{1}{\ln(d!)}\sum_{k=1}^{d!}p^{*}(\pi_{k})\cdot \ln(p^{*}(\pi_{k}))\text{​}$$

The value of d is crucial to the calculation of PE and AAPE. The larger d, the more reliable results are usually obtained. But if d is too large, it takes longer to calculate the entropy. In this paper, based on the length of time series analyzed, we set d=5.

In order to more intuitively verify and compare the performances of the PE and the AAPE, some quintessential simulation signals are given. The simulation signals $f_{1}$, $f_{2}$, and $f_{3}$ can be determined by:

(12) $$\left\{ \begin{array}{l} s_{1}=0.5\cos(50\pi n),0\leq n\leq 1\\ s_{2}=0.9\cos(250\pi n),0.3\leq n\leq 0.7\\ f_{1}=s_{1}+s_{2}\\ f_{2}=s_{1}+3s_{2}\\ f_{3}=s_{1}+s_{2}+1.5 \end{array}\right.$$

where $s_{1}$ and $s_{2}$ are two components of $f_{1}$, $f_{2}$, and $f_{3}$, respectively, and the sampling frequency is 1000 Hz. The PEs and AAPEs of the three simulated signals are listed in Table 1.

As shown in Table 1, for three different simulated signals, the PEs are completely equal and the AAPEs are different. The results of this study indicate that PE cannot distinguish three signals in this case accordingly, and AAPE can better measure time series complexity.

### 2.3. Pearson Correlation Coefficient (PCC)

Pearson correlation coefficient (PCC) is an indicator used to quantify the linear relation between two stochastic variables, X and Y. In order to determine whether the high-frequency IMF with the smallest AAPE is a UIMF, this paper uses PCC to calculate the similarity between the IMF and the original signal. Then the PCC can be expressed as follows:

(13) $$PCC(X,Y)=\frac{E[(X-\mu_{X})(Y-\mu_{Y})]}{\sigma_{X}\sigma_{Y}}$$

where $\mu_{X}$ is the mean of the variable *X*, $\mu_{Y}$ is the mean of the variable *Y*, $\sigma_{X}$ is the standard deviation of *X* and $\sigma_{Y}$ is the standard deviation of *Y*. The correlation coefficient can range between −1 and +1 and reveals two aspects about the linear relation between variables: its strength and direction. The direction is determined by the sign, which indicates whether the variables are positive correlated or anti-correlated [35]. The relationship between PCC and correlation is given in Table 2.

## 3. The Proposed Noise Reduction Method

### 3.1. The Proposed Noise Reduction Method

**Figure 1:**
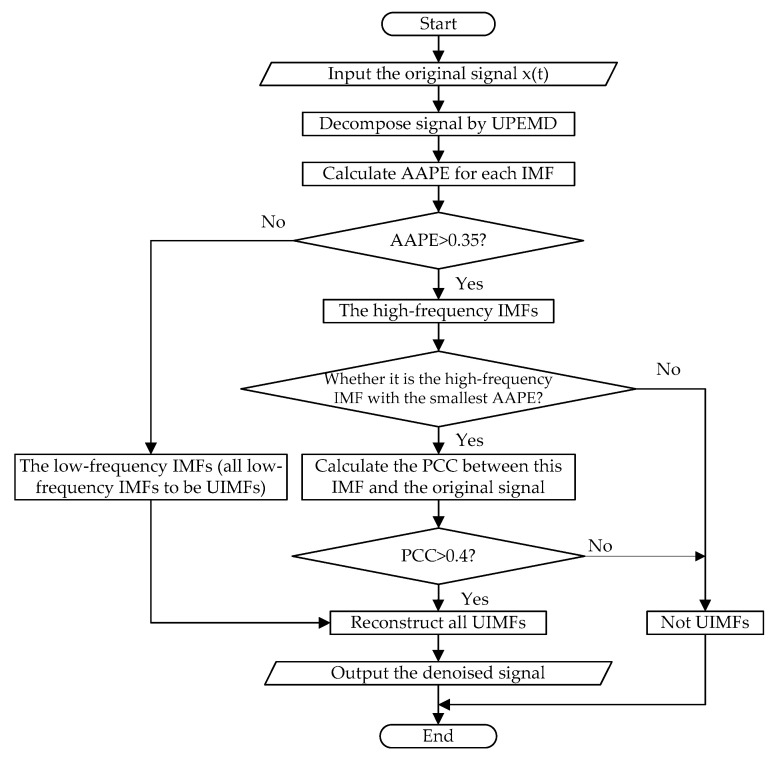
The flow chart of the proposed noise reduction method.

The flow chart of the proposed noise reduction method (namely UPEMD-AAPE-PCC) is shown in Figure 1.

The specific steps of the proposed method are as follows:

Step 1. Decompose the original signal using UPEMD.
Step 2. Calculate the AAPE of each IMF.
Step 3. Determine the threshold of AAPE. These IMFs, where AAPE is less than a given threshold, are determined as low-frequency IMF (all low-frequency IMFs to be UIMFs). The remaining IMFs are determined as high-frequency IMFs. When the embedded dimension of AAPE is 5, it is appropriate to set the threshold to 0.35, which will be proved in Section 4.1.
Step 4. Calculate PCC between the high-frequency IMF with the smallest AAPE and the original signal. If PCC is greater than 0.4, the IMF is also determined as a UIMF.
Step 5. Reconstruct all UIMFs. After the reconstruction, the process of noise reduction is completed.

### 3.2. Evaluation Criteria for Chaotic Signal Noise Reduction

In order to evaluate the performance of the proposed noise reduction method, the proposed method is applied to the denoising experiment of chaotic signal. For the sake of evaluating the performance of these methods, the signal-to-noise ratio (SNR) and root mean square error (RMSE) are adopted and defined as follows [36]:

(14) $$SNR=10\cdot \log_{10}\left(\frac{{\left\|x\right\|}^{2}}{{\left\|\hat{x}-x\right\|}^{2}}\right)$$

(15) $$RMSE=\sqrt{\frac{{\left\|\hat{x}-x\right\|}^{2}}{N}}$$

where x and $\hat{x}$ are the noiseless signal and the denoised signal; N represents the length of the signal.

### 3.3. Evaluation Criteria for Underwater Acoustic Signals Noise Reduction

For the underwater acoustic signals without prior knowledge, the dynamical behavior of denoised signal is analyzed with noise intensity [1], correlation dimension [37] and spatial-dependence recurrence sample entropy (SdrSampEn) [38] to show the validity of the proposed method. Since the original signal contains a multitude of noises, these feature parameters will be larger than the denoised signal, and we use these feature parameters to evaluate the noise reduction effect.

#### 3.3.1. Noise Intensity

Assuming a time series of N samples in length, that is x(n)={x(1),x(2),…,x(N)}. Its noise intensity can be approximated by the standard deviation, i.e.,:

(16) $$\sigma =\sqrt{\frac{1}{N}\sum_{n=1}^{N}{\left[x(n)-\bar{x}\right]}^{2}}$$

where $\bar{x}=\frac{1}{N}\sum_{n=1}^{N}x(n)$ represents the mean of the time series.

#### 3.3.2. Correlation Dimension

The given time series of a dynamic system $x_{k}=\;x(k\Delta t)$, (k=1,2,…,N) is embedded into a *m*-dimensional space to fully expose the information contained in the time series [37]. For this phase space, it is converted into a set of vectors as follows:

(17) $$X_{n}(m,\tau )=\{x_{n},x_{n+k},\ldots ,x_{n+(m-1)k}\}$$

where τ=kΔt is the time delay, Δt is the sampling interval, $X_{n}$ is the N vector in *m*-dimensional space, n=1,2,…,M, and M=N−(m−1)τ is the total number of vectors in reconstructed *m*-dimensional phase space.

Thus, the reconstructed phase space is defined as:

(18) $$X={\left[X_{1}(m,\tau ),X_{2}(m,\tau ),\ldots ,X_{M}(m,\tau )\right]}^{T}$$

First we calculated the distance between all the vectors in the phase space. We define the phase point $X_{i}$, which is chosen arbitrarily from the M data points, as the reference phase point and calculated the spatial distance $d_{ij}$ between $X_{i}$ and the other M−1 phase points:

(19) $$d_{ij}=\left\|X_{i}-X_{j}\right\|$$

where $X_{i}$ and $X_{j}$ are the two points in the phase space reconstruction.

After calculating all the points, the correlation integral can be obtained as:

(20) $$C(r)=\frac{2}{M(M-1)}\sum \theta (\varepsilon -d_{ij})$$

where r>0, and 1≤i≤j≤M.

C(r) represent the percentage of the number of point pairs in the phase space whose radius is smaller than r, and θ(x) is the Heaviside function, which is defined as:

(21) $$\theta (x)=\{\begin{array}{c} 0,x<0\\ 1,x\geq 0 \end{array}$$

Therefore, the correlation dimension $D_{2}$ is calculated by the correlation integral C(r) and the radius r with:

(22) $$D_{2}=\frac{d\ln C(r)}{d\ln r}$$

$D_{2}$ is regarded as the slope of the lnC(r)-lnr line segments when r is in a relatively small value and the correlation of lnC(r) with lnr is indicated approximately straight. With a growing m, $D_{2}$ increases and becomes saturated gradually. It is worth noting that the calculation of the correlation dimension is more reliable when the length of the data is large enough [39].

#### 3.3.3. Spatial-Dependence Recurrence Sample Entropy (SdrSampEn)

SdrSampEn is based on the mathematical principle of sample entropy and enables the capture of sequential information of a time series in the context of spatial dependence provided by the binarylevel co-occurrence matrix of a recurrence plot. Pham et al. [38] used SdrSampEn to measure the uncertainty of Lorenz signal. At the same time, it is pointed out that SdrSampEn have a better discriminative ability in measuring the irregularity of chaotic time series. Detailed calculation of SdrSampEn can be seen in [38].

## 4. The Chaotic Signal Denoising Experiment

Underwater acoustic signals have obvious chaotic characteristics. Three quintessential chaotic signals are selected for simulation experiments, including Lorenz signal, Rossler signal, and Duffing signal. In order to show the superiority of the method, EMD-AAPE-PCC, EMSD-AAPE-PCC and the proposed method are used for comparison.

### 4.1. Choice the Threshold of AAPE

After decomposing the original signal into a series of IMFs, and determining whether they are UIMFs in accordance with AAPE, we need to set the corresponding threshold. Therefore, it is indispensable to study the threshold of AAPE. For the sake of brevity, this section selects the Lorenz signal to analyze.

**Figure 2:**
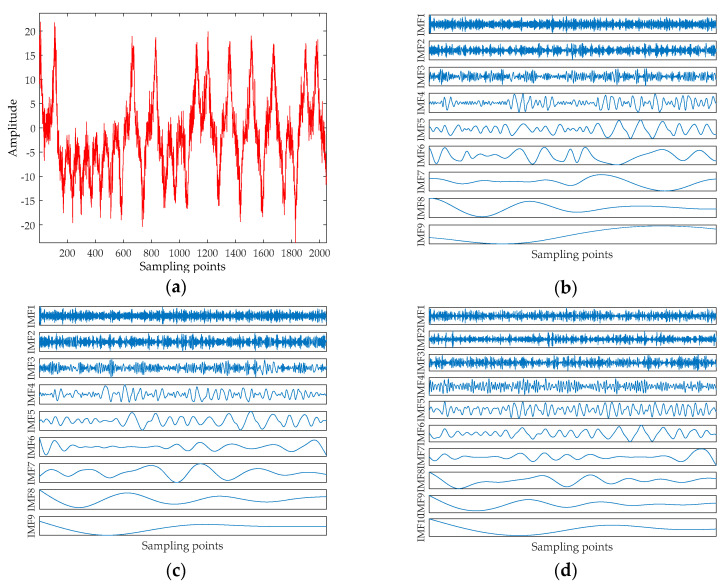
The noisy Lorenz signal and the decomposition result of EMD, ESMD and UPEMD. (**a**) The noisy Lorenz signal; (**b**) The decomposition result of EMD; (**c**) The decomposition result of ESMD; and (**d**) The decomposition result of UPEMD.

The Lorenz system can be expressed as:

(23) $$\begin{array}{l} \dot{x}=-\sigma (x-y)\\ \dot{y}=-xz+rx-y\\ \dot{z}=xy-bz \end{array}$$

where σ=10, b=8/3, r=28. The equation is integrated by using a fourth-order Runge–Kutta method with a fixed step size of 0.01, and the parameter values of the equation are x=−1, y=0 and z=1. The x component signal with a length of 2048 points is selected as a chaotic signal, and the signal are added the Gaussian white noise with SNR = 10 dB. The time-domain waveform for noisy Lorenz signal are shown in Figure 2a. The decomposition result of the EMD, ESMD, UPEMD for the noisy Lorenz signal are presented in Figure 2b–d.

**Figure 3:**
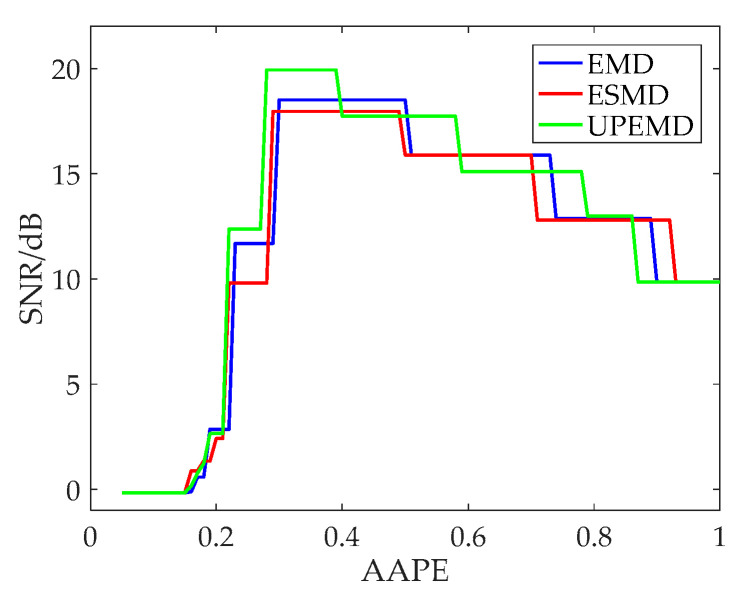
The SNR with different AAPE thresholds.

As can be seen in Figure 2, EMD and ESMD decompose the original signal into 9 IMFs, and UPEMD decomposes the original signal into 10 IMFs. The AAPE of each IMF is listed in Table 3. In order to choose the appropriate AAPE threshold, we set the threshold from 0.1 to 1 and the increment is 0.01. When the AAPE is less than this value, the corresponding IMFs are added to obtain a recombined signal. Finally, the SNR of the recombined signal is shown in Figure 3.

As indicated in Figure 3, as the AAPE increases, the SNR first increases to a certain extent and then decreases. When the AAPE is around 0.3, the SNR of the three decomposition algorithms reaches the maximum for the first time. As AAPE continues to increase, the SNR of UPEMD begins to decline around 0.4, and the SNR of EMD and ESMD begins to decrease around 0.5. After testing, we found that the threshold selection presented similar results for the Duffing signal and the Rossler signal, which are not listed here. For convenience, this paper sets the AAPE threshold to 0.35.

### 4.2. Denoising for Noisy Chaotic Signal

**Figure 4:**
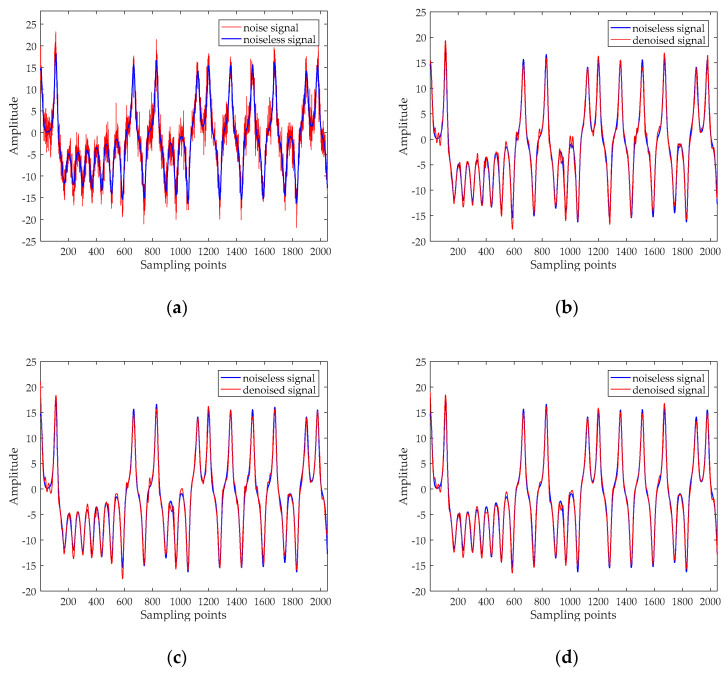
The time-domain waveform before and after noise reduction for noisy Lorenz signal when SNR is 10 dB. (**a**) The time-domain waveform of the noisy signal and noiseless signal; (**b**) the time-domain waveform after noise reduction by EMD-AAPE-PCC; (**c**) the time-domain waveform after noise reduction by ESMD-AAPE-PCC; and (**d**) the time-domain waveform after noise reduction by the proposed method.

**Figure 5:**
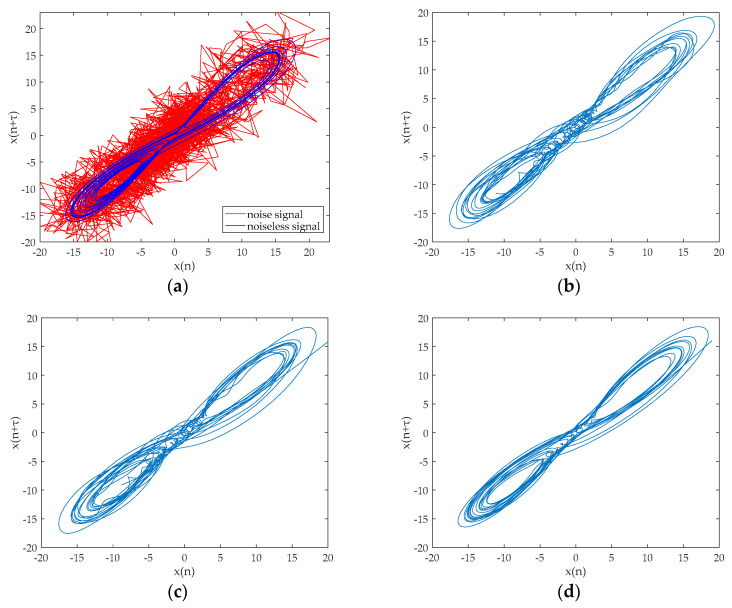
The phase diagram before and after noise reduction for noisy Lorenz signal when SNR is 10 dB. (**a**) The phase diagram of noisy signal and noiseless signal; (**b**) the phase diagram after noise reduction by EMD-AAPE-PCC; (**c**) the phase diagram after noise reduction by ESMD-AAPE-PCC; and (**d**) the phase diagram after noise reduction by the proposed method.

This section also selects the Lorenz signal to analyze, and the test results of the Rossler signal and the Duffing signal will be given later. The x component signal with a length of 2048 points is selected as a chaotic signal, and the signal is added the Gaussian white noise with different SNRs. The time-domain waveform before and after noise reduction for noisy Lorenz signals when SNR = 10 dB are shown in Figure 4, and their phase diagrams are shown in Figure 5. The noise reduction results for chaotic signal (including Lorenz signal, Rossler signal and Duffing signal with SNR = 5 dB, 10 dB, 15 dB, and 20 dB are listed in Table 4.

Figure 4 revealed that there are still some noises based on the EMD and ESMD methods, the noise reduction result of UPEMD has the highest similarity with the noiseless signal. In order to better prove the noise reduction effect of the three methods, we compare the phase diagrams before and after noise reduction. As presented in Figure 5a, the phase diagram of the noisy Lorenz signal is annihilated by noise and appears as a disorderly pseudo-random feature. As can be seen from Figure 5d, the phase diagram has a smoother track after noise reduction using UPEMD. Compared with EMD and ESMD, we can see that the denoised phase diagram of UPEMD is closest to the noiseless signal. Table 4 presents the comparison of the SNR and the RMSE of the above three methods. Compared with EMD-AAPE-PCC and ESMD-AAPE-PCC, the proposed method further enhances the SNR and reduces the RMSE. It is obvious that the method in this paper has improving the performance.

## 5. The Underwater Acoustic Signals Denoising Experiment

### 5.1. Data Collection

In this paper, three different types of ship radiated noise are selected as sample data, namely the Ship-I, the Ship-II, and the Ship-III. The ship radiated noise are measured in the South China Sea. Each type of underwater acoustic signals has 100 sample data. Each sample length is 2048 points and sampling interval is 0.05 ms. Before the noise reduction experiment, the sample data have been filtered, normalized, and sampled.

### 5.2. Denoising for Underwater Acoustic Signals

**Figure 6:**
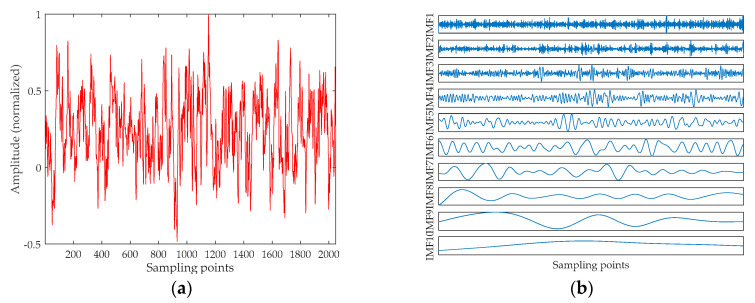
The time-domain waveform of the Ship-I and the decomposition result of UPEMD. (**a**) The time-domain waveform of the Ship-I; and (**b**) the decomposition result of UPEMD.

**Figure 7:**
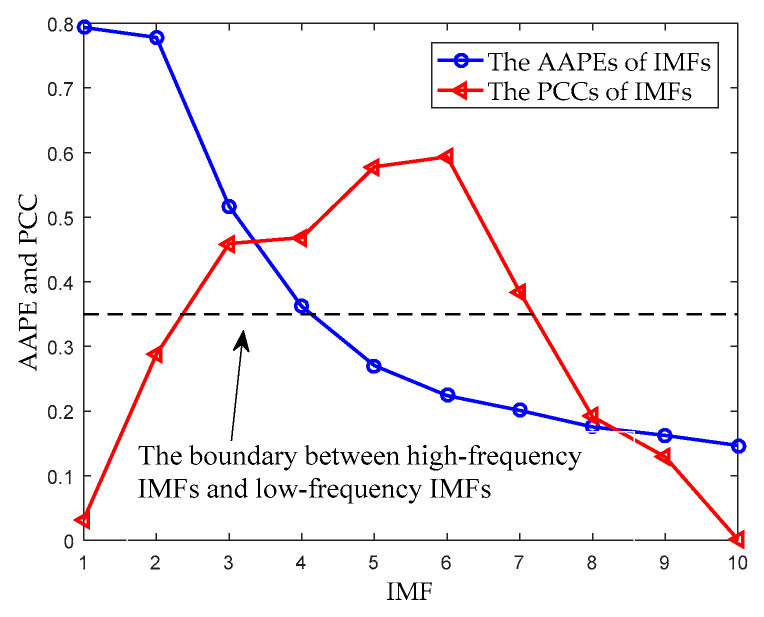
The SNR with different AAPE thresholds.

For the sake of brevity, this section selects the Ship-I to analyze. The time-domain waveform of the Ship-I is shown in Figure 6a. The decomposition result of UPEMD for the Ship-I is presented in Figure 6b. The AAPEs and PCCs of these components are indicated in Figure 7.

**Figure 8:**
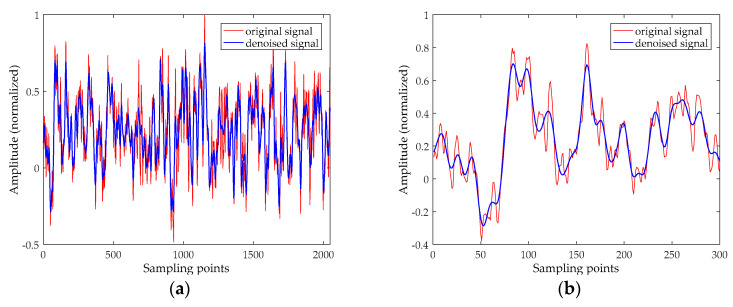
The time-domain waveform before and after noise reduction for the Ship-I. (**a**) The time-domain waveform before noise reduction; and (**b**) the time-domain waveform before noise reduction (select 300 points).

**Figure 9:**
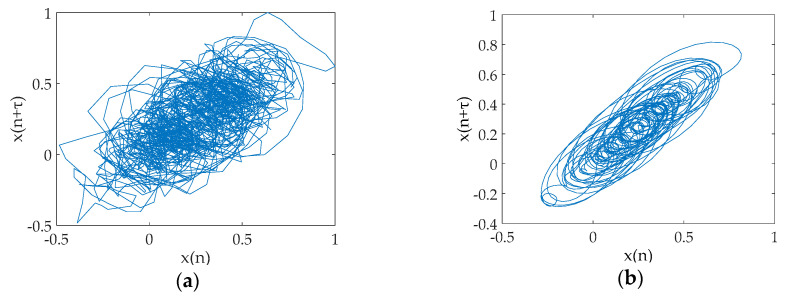
The phase diagram before and after noise reduction for the Ship-I. (**a**) The phase diagram before noise reduction; and (**b**) the phase diagram after noise reduction.

From the AAPE curve in Figure 7, the proposed method determines the high-frequency IMFs and low-frequency IMFs by AAPE, and the AAPE decreases as the IMF order increases. The AAPEs of the last six IMFs (IMF5~IMF10) are all less than 0.35, and they are identified as low-frequency IMFs. From the PCC curve, we find the high-frequency IMF with the smallest AAPE, which is IMF4. It can be found that the PCC between IMF4 and the original signal is greater than 0.4, consequently, IMF4 is also determined as a UIMF. The time-domain waveforms before and after noise reduction of the Ship-I are shown in Figure 8. The phase diagrams before and after noise reduction are shown in Figure 9.

**Figure 10:**
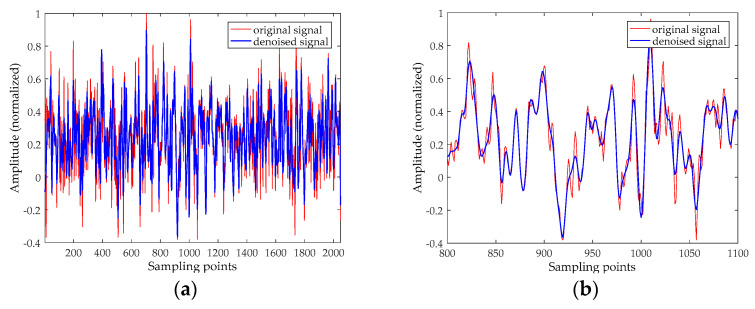
The time-domain waveform before and after noise reduction for the Ship-II. (**a**) The time-domain waveform before noise reduction; and (**b**) the time-domain waveform before noise reduction (select 300 points).

**Figure 11:**
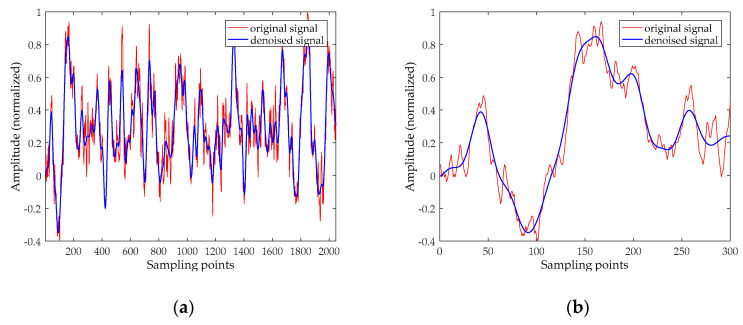
The time-domain waveform before and after noise reduction for the Ship-III. (**a**) The time-domain waveform before noise reduction; and (**b**) the time-domain waveform before noise reduction (select 300 points).

**Figure 12:**
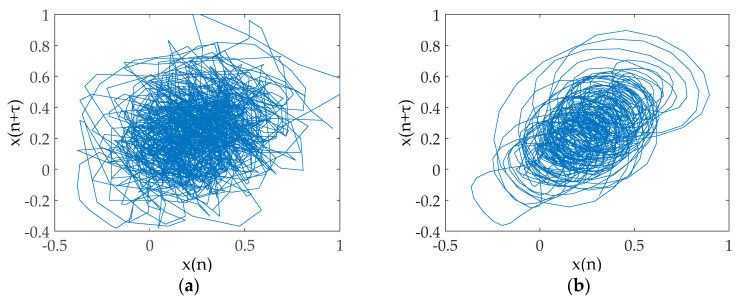
The phase diagram before and after noise reduction for the Ship-II. (**a**) The phase diagram before noise reduction; and (**b**) the phase diagram after noise reduction.

**Figure 13:**
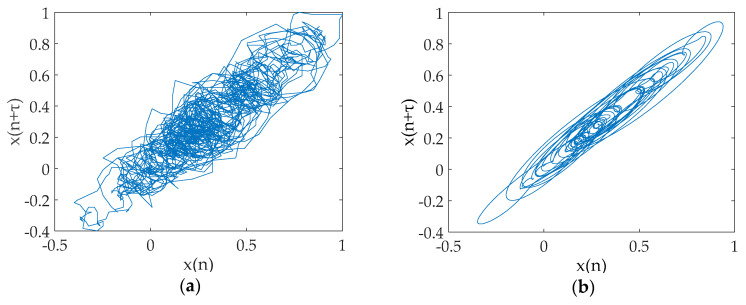
The phase diagram before and after noise reduction for the Ship-III. (**a**) The phase diagram before noise reduction; and (**b**) the phase diagram after noise reduction.

Using the same method, we perform noise reduction on the Ship-II and the Ship-III. The time-domain waveforms before and after noise reduction are presented in Figure 10 and Figure 11, respectively. The phase diagrams before and after noise reduction are presented in Figure 12 and Figure 13, respectively. In order to measure the effect of the noise reduction method for real underwater acoustic signals, we calculate some feature parameters and make a comparison before and after noise reduction, as listed in Table 5.

It can be seen from Figure 8, Figure 10 and Figure 11 that underwater acoustic signals before noise reduction is full of a great deal of noise, and it is completely infeasible to distinguish the trend of the waveform. After noise reduction, the noises are well suppressed. Figure 9, Figure 12 and Figure 13 indicated that the phase diagrams of underwater acoustic signals are obliterated by noise before noise reduction, and fractal characteristics are almost not observed. After noise reduction, the proposed method can roughly restore the phase diagram of the pure attractor. The denoised signal has a smoother orbit, and the attractor has better geometric regularity. As demonstrated in Table 5, the denoised signal has smaller noise intensity, correlation dimension, and SdrSampEn, indicating that the method removes most of the noises in the underwater acoustic signals, further reflecting the effectiveness of the proposed method.

## 6. Conclusions

In this paper, a new noise reduction method for underwater acoustic signals based on UPEMD, AAPE, and PCC is proposed. The proposed method uses UPEMD to decompose the original signal, distinguishes the high-frequency and low-frequency IMFs by AAPE, judges whether the high-frequency IMF with the smallest AAPE is a UIMF according to PCC, and finally reconstructs all UIMFs. The main findings in this paper are highlighted as follows:

(1) UPEMD, as a new adaptive decomposition algorithm, is first used in the noise reduction of underwater acoustic signals.
(2) A simulation experiment shows that AAPE can reflect the amplitude information of the signal compared with PE. Consequently, AAPE is used to measure the complexity of IMF in this paper.
(3) Quantitative comparisons based on the noisy chaotic signals demonstrate that the proposed method performs better than the EMD-AAPE-PCC and the ESMD-AAPE-PCC method by providing lower RMSE and higher SNR value.
(4) Through the noise reduction experiments of three types of underwater acoustic signals, it is proved that the proposed method can further eliminate the noise and recover the true dynamic characteristics of the chaotic signal more clearly, which lays a foundation for the study of the detection, feature extraction and classification of underwater acoustic signals.

## Figures and Tables

**Table 1:** The PEs and AAPEs of simulated signals.

Parameter	$f_{1}$	$f_{2}$	$f_{3}$
PE	0.6513	0.6513	0.6513
AAPE	0.6879	0.7214	0.6403

**Table 2:** The relationship between PCC and correlation.

Parameter	No Correlation	Weak Correlation	Moderate Correlation	Strong Correlation
PCC	0~0.1	0.1~0.3	0.3~0.5	0.5~1

**Table 3:** The AAPEs of IMFs.

Method	IMF1	IMF2	IMF3	IMF4	IMF5	IMF6	IMF7	IMF8	IMF9	IMF10
EMD	0.8990	0.7361	0.5074	0.2929	0.2236	0.1896	0.1642	0.1621	0.1512	/
ESMD	0.9230	0.7038	0.4938	0.2810	0.2185	0.1932	0.1742	0.1600	0.1533	/
UPEMD	0.8681	0.7870	0.5886	0.3978	0.2704	0.2188	0.1895	0.1737	0.1614	0.1506

**Table 4:** Noise reduction results of chaotic signal.

Chaotic Signal	SNR/dB	EMD-AAPE-PCC		ESMD-AAPE-PCC		UPEMD-AAPE-PCC	
		SNR/dB	RMSE	SNR/dB	RMSE	SNR/dB	RMSE
Lorenz signal	5	13.7972	1.5807	13.3847	1.6575	15.6321	1.2797
	10	19.5190	0.8180	19.2559	0.8431	21.0471	0.6860
	15	23.3469	0.5264	21.8157	0.6279	25.5440	0.4088
	20	26.2986	0.3748	26.1920	0.3794	29.1438	0.2701
Rossler signal	5	13.8541	0.9662	14.4716	0.8999	17.3298	0.6475
	10	18.5442	0.5630	18.4306	0.5705	20.7218	0.4382
	15	23.9379	0.3026	22.9977	0.3372	26.1470	0.2346
	20	28.6691	0.1755	28.8895	0.1711	30.4150	0.1435
Duffing signal	5	13.6513	0.4619	13.9917	0.4442	17.0769	0.3114
	10	18.6499	0.2598	18.6051	0.2611	20.4668	0.2108
	15	22.5043	0.1667	21.6795	0.1833	24.1256	0.1383
	20	18.6596	0.2595	26.5844	0.1042	26.9542	0.0999

**Table 5:** Feature parameters before and after noise reduction.

Underwater Acoustic Signals	Status	Noise Intensity	Correlation Dimension	SdrSampEn
The Ship-I	Before noise reduction	0.2318	2.1261	1.7626
	After noise reduction	0.2034	1.5841	0.5991
The Ship-II	Before noise reduction	0.2105	2.5325	2.9703
	After noise reduction	0.1799	1.8446	1.1416
The Ship-III	Before noise reduction	0.2561	1.9459	0.9726
	After noise reduction	0.2388	1.2441	0.3647
